# Supplementary material for: Formation of recurring transient Ca2+-based intercellular communities during Drosophila hematopoiesis
Source: Proc Natl Acad Sci U S A. 2024 Apr 11;121(16):e2318155121. doi: 10.1073/pnas.2318155121 (PMC11032476; doi:10.1073/pnas.2318155121)
Supplement: Supplementary file 1 — Appendix 01 (PDF) [file pnas.2318155121.sapp.pdf]

Supplemental Figure titles and legends

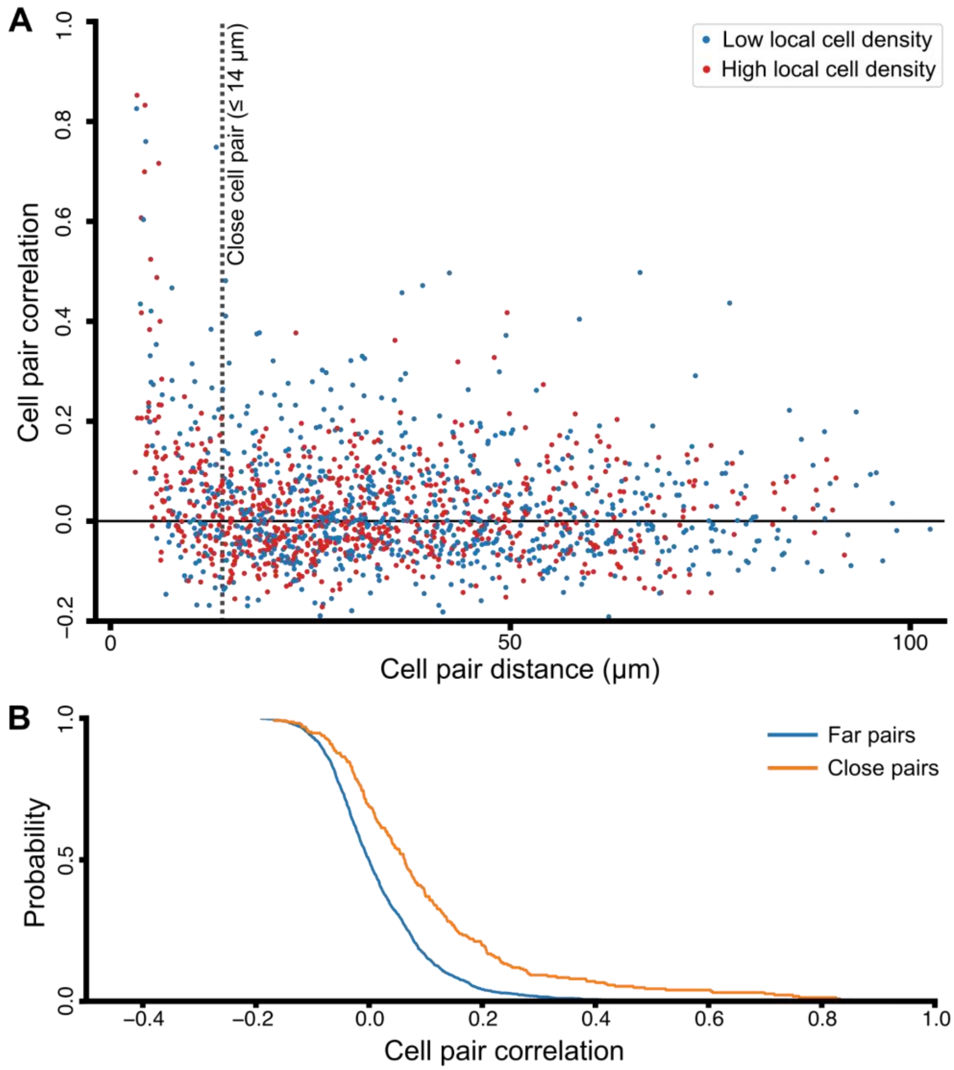

Figure S1. Close blood progenitor pairs are more synchronized in their Ca2+ activities than distant cell pairs. (A) Spatial analysis of all blood progenitor pairs from the same LG as Fig 1B. The cell pair correlations were associated with the cell pair distances. Each data point represents a cell pair and color represents low (blue) or high (red) local cell density. N_{cells}= 57, N_{pairs}= 1596, Pearson correlation between the Ca^{2+} correlation of cell pairs and their corresponding distance = -0.15, p-value < 0.0001. The local cell density was determined as the mean count of cell centers within a radius of 7µm around each cell's center within the pair. Local cell density of 1-3.25was categorized as low-density (blue), and local cell density exceeding 3.25 was categorized as high-density (red). Pearson correlation between the Ca^{2+} correlation of cell pairs and their corresponding distances was not sensitive to local cell density: $Correlation_{low}$ = -0.157, P_{low} < 0.0001, $Correlation_{high}$ = -0.155, P_{high} < 0.0001. **(B)** Cumulative distribution of Pearson correlation of the close (orange; N=227, µ=0.101, σ=0.181) and far (blue; N=1369, µ=0.016, σ=0.097) blood progenitor pairs (same pairs as in A). Each value $F_g(x)$ in the plot is the probability of a pair in group g having a Pearson correlation coefficient greater than x. Kruskal-Wallis statistical test verified a significant difference between the two distributions (p-value < 0.0001).

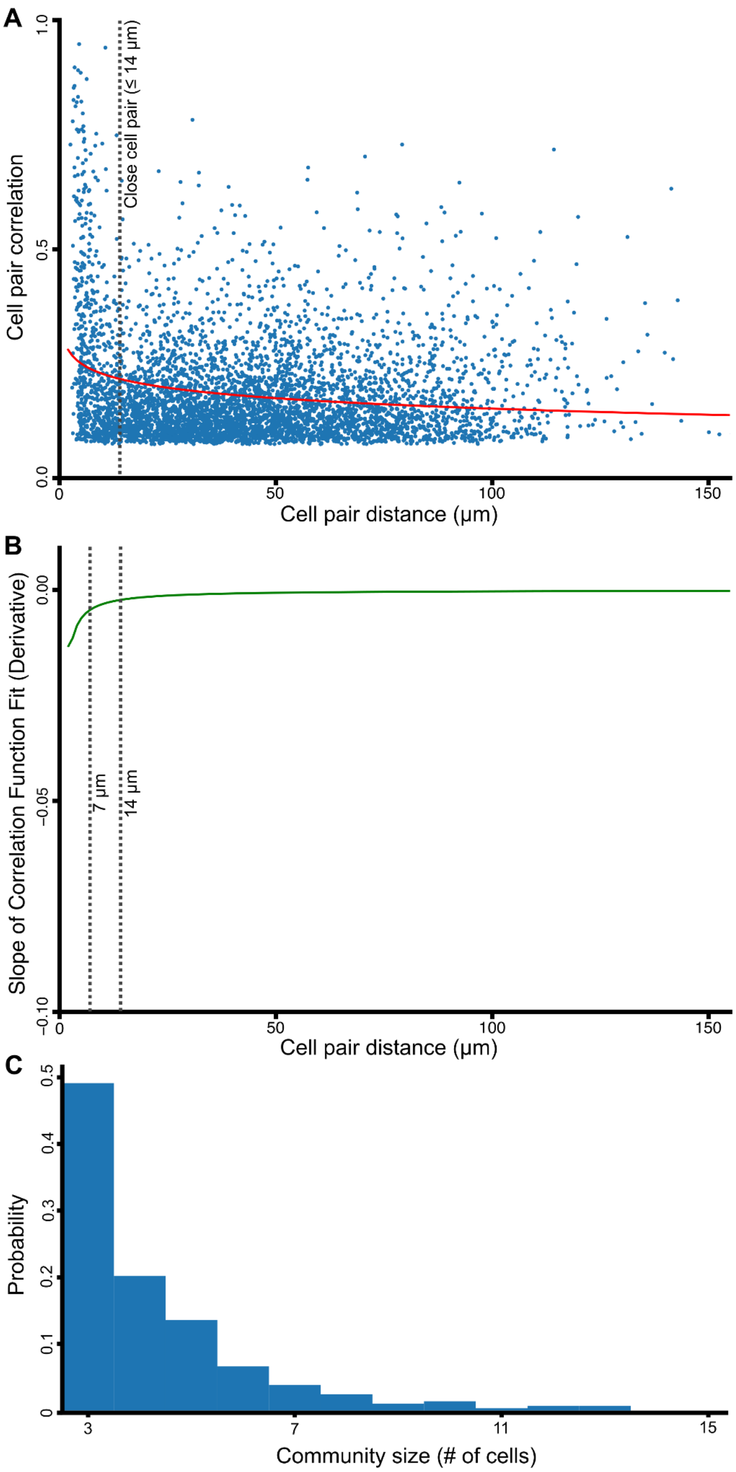

Figure S2. Community detection using ARCOS. (A) Spatial analysis of all blood progenitor pairs pooled across all 12 wild-type LGs. The cell pair correlations were associated with the cell pair distances. Each data point represents a cell pair (from the same LG). N_{cells}= 722, N_{pairs}= 4957. The logarithmic fit of the cell pairs correlation as a function of the cell pair distances (red line). **(B)** The derivative of the logarithmic fit from panel A, that is, the change in the correlation as a function of the pair distance (green). The dashed vertical line at 14 μm (approximately two cell diameters) marks the point at which

the logarithmic function completed 85% of its decay, displayed minimal further change and thus was selected as the spatial parameter for the ARCOS analysis. **(C)** Transient communities pooled across 12 wild-type LGs. Each bar is the probability of a community involving the corresponding number of cells. $N_{communities} = 288$, $\mu_{size} = 4.302$.

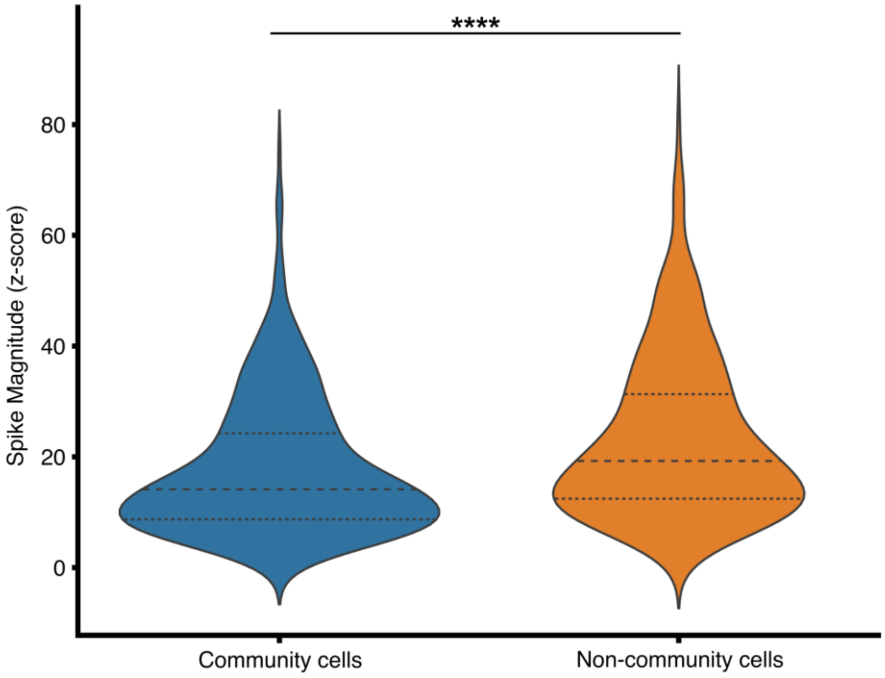

Figure S3. Cells participating in communities exhibit lower Ca^{2+} spike magnitudes than cells that do not. Distribution of the Ca^{2+} signal spike magnitude of cells participating in communities (blue) and those that did not participate in communities (orange). The Ca^{2+} magnitude was calculated for each cell as the average z-score of its Ca^{2+} peaks in respect to its time-series background Ca^{2+} signal (see Methods). Kruskal-Wallis statistical test verified a significant difference between the two distributions ($p\text{-value} < 0.0001$), with a median z-score of 14.143 versus 19.253 for community and non-community cells respectively. Cells were pooled across all 12 wild-type LGs.

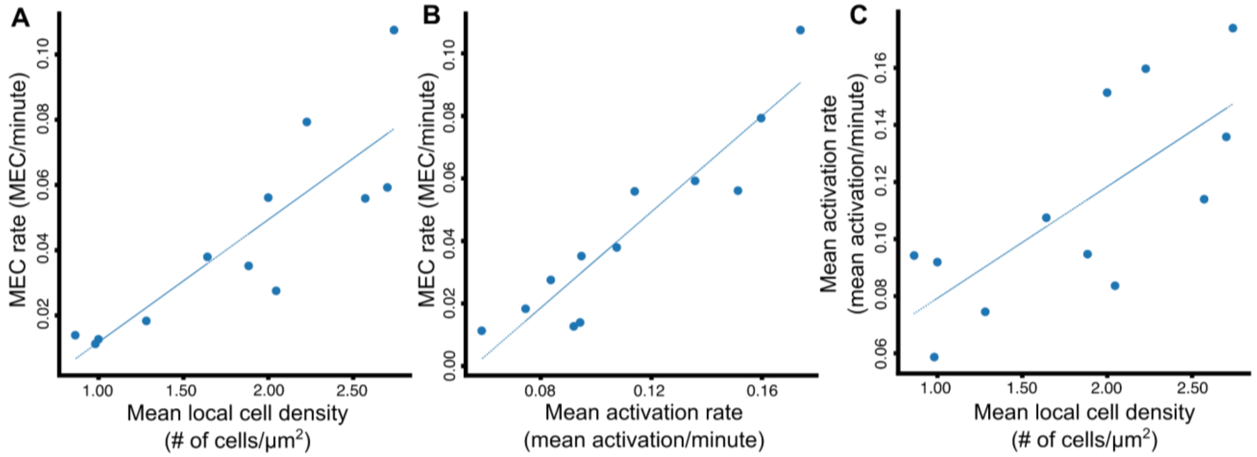

Figure S4. Correlation between potential confounding factors. Each data point reflects the mean value of the corresponding measurements across all cells in an experiment, N = 12 wild-type LGs. MEC rate is the number of mean events per cell per minute. The mean activation rate is the average number of activations per cell per minute. Mean local cell density is the average number of cells within an area of 14x14 μm^2 surrounding each cell. The line represents linear fit. **(A)** Pearson correlation (coefficient = 0.854, p-value = 0.0004) between MEC rate and the mean local cell density **(B)** Pearson correlation (coefficient = 0.929, p-value < 0.0001) between MEC rate and mean activation rate. **(C)** Pearson correlation (coefficient = 0.735, p-value = 0.0065) analysis between mean activation rate and mean local cell density.

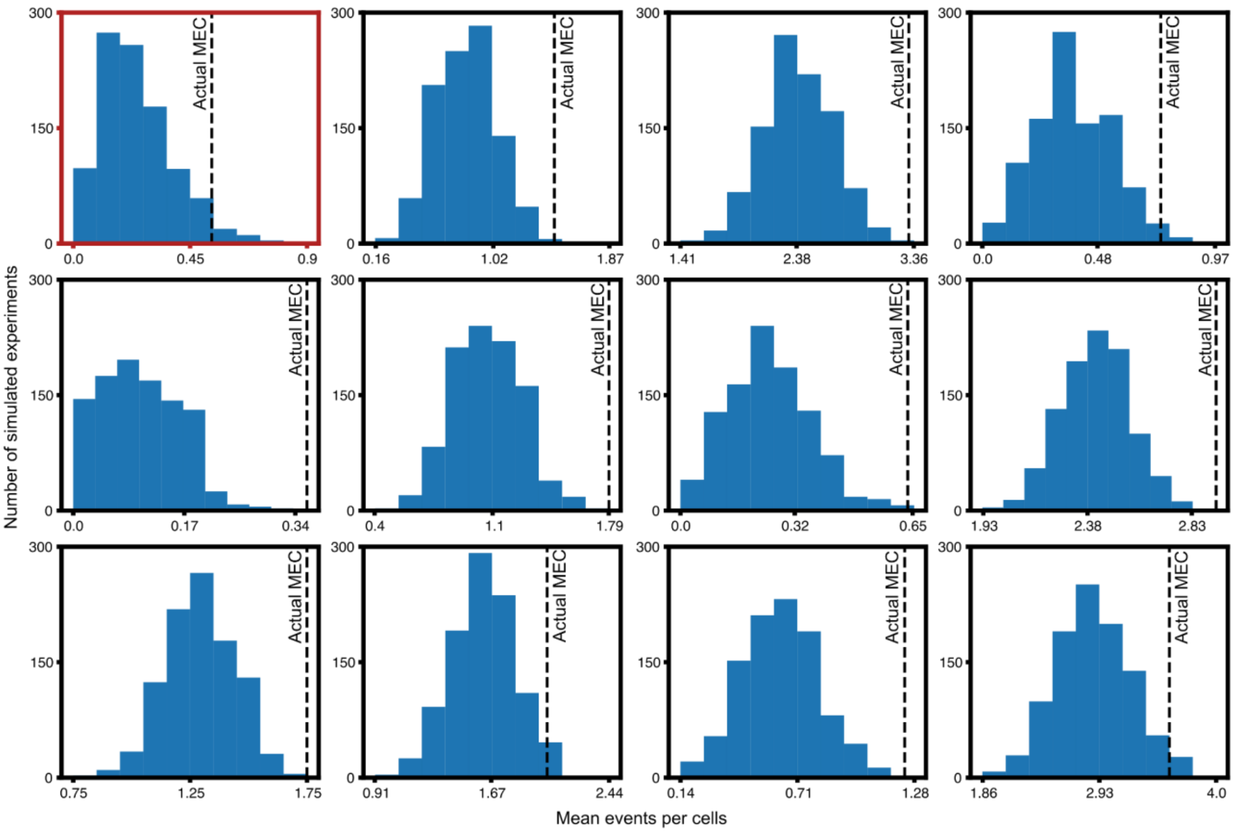

Figure S5. Mean events per cell (MEC) spatial shuffling analysis of wild-type LGs. Histograms of the MECs for 1000 *in-silico* spatially permuted wild-type LG. Vertical dashed line marks the actual MEC observed in the experiment. Top left histogram (red) refers to the single spatially insignificant wild-type LG from Fig. 1F and 1G, which achieved a "nearly" significant p-value of 0.059. Note that this LG had the least number of cells (n = 30) we could measure from, and the least number of transient communities (n = 5) among all wild-type LGs making the statistical analysis more sensitive.

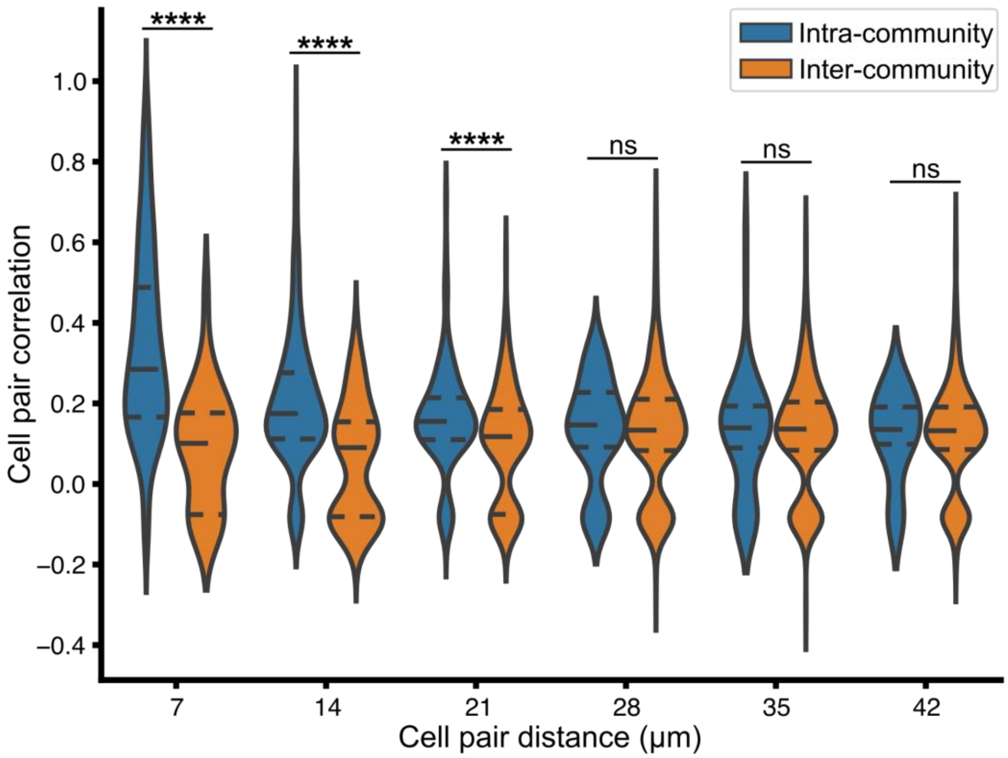

Figure S6. Elevated intra- versus inter-community cell pair correlations. Comparison of cell pair correlations within the same transient community ("intra-community") and between different communities ("inter-community"). Each cell pair's maximal cross-correlation was recorded, allowing a time lag of up to 15 seconds, in accordance with the temporal parameter used in ARCOS to define transient communities. Pairs were grouped according to their cell pair distance, with 7μm intervals (approximately 1 cell diameter). Kruskal-Wallis test confirmed that intra-community pairs showed higher correlation than inter-community pairs for distances up to 21μm (p-value < 0.0001). Specifically, the median correlation was 0.284 vs. 0.100 for pairs 0-7μm apart, 0.174 vs. 0.090 for 7-14μm, and 0.155 vs. 0.117 for 14-21μm, intra- vs. inter-pairs, respectively.

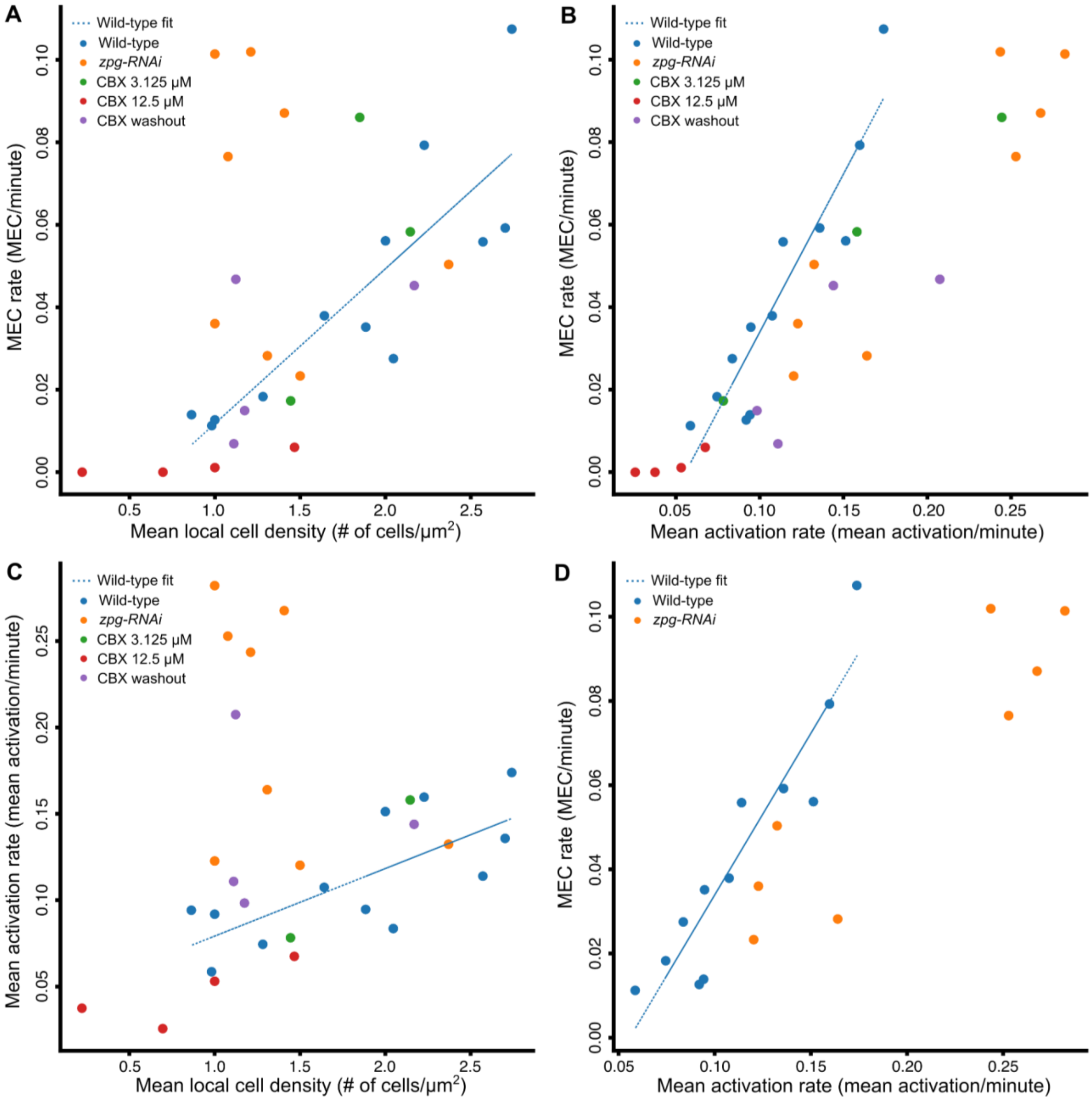

Figure S7. Correlation between potential confounding factors in gap junction inhibition experiments. Each data point reflects the mean value of the corresponding measurements across all cells in an experiment, with wild-type LGs in blue (N=12), RNAi-mediated knockdown of *zpg* in orange (N=8), 3.125 µM CBX in green (N=3), 12.5 µM CBX in red (N=4), and CBX washout in purple (N=4). The blue line represents the linear fit based on the wild-type LGs. **(A)** Pearson correlation (coefficient = 0.487, p-value = 0.0055) between MEC rate and the mean local cell density. (**B**) Pearson correlation (coefficient = 0.876, p-value < 0.0001) between MEC rate and mean activation rate. **(C)** Pearson correlation (*zpg RNAi* data was excluded from the calculation of the Pearson correlation, see panel D; coefficient = 0.573, p-value = 0.0042) between mean activation rate and mean local cell density. **(D)**

MEC rate and mean activation rate for wild-type (blue; N=12) and RNAi-mediated knockdown of *zpg* (orange; N=8) LGs. Data as in panel B. The distance of each *zpg RNAi* LG from the wild-type linear fit was calculated as the subtraction between the observed and the corresponding linear-fit MEC rate ($\mu_{wild\ type} = (-9.83e)^{-18}$, $\sigma_{wild\ type} = 0.01$, $\mu_{RNAi\ zpg} = -0.046$, $\sigma_{RNAi\ zpg} = 0.025$). Kruskal-Wallis statistical test verified a significant difference between the distance distributions of the two experimental groups (p-value = 0.0009).

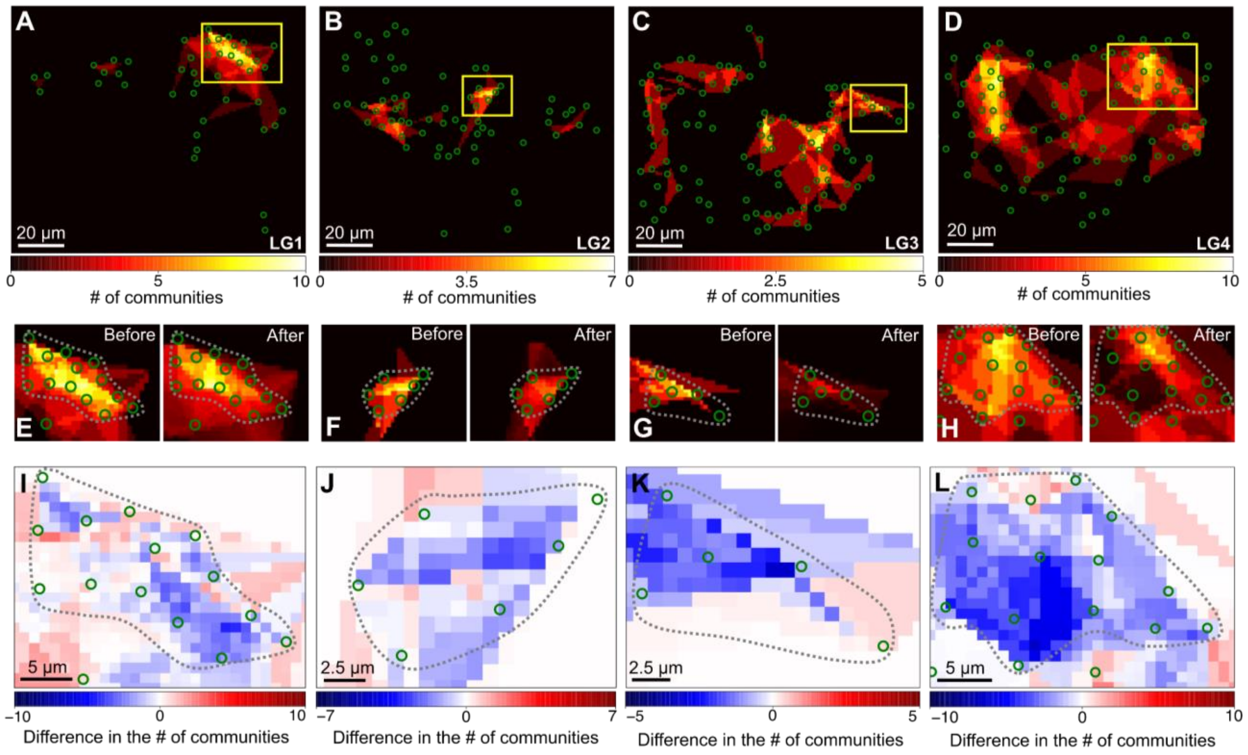

Figure S8. Spatial *in silico* permutations of hotspot cells reduce communication in a hotspot. (A-D) Visualization of the integrated number of transient communities each cell participated in over time in different wild-type LGs. Green circles: the center of each blood progenitor. Color-coded legend: the number of communities. Yellow regions of interest mark hotspots. **(E-H)** Hotspots (dashed lines) before and after *in silico* permutation. Each hotspot corresponds to the hotspot above it in panels A-D **(I-L)** The difference in the number of transient communities within a hotspot (dashed line) after *in silico* permutations, negative values indicate reduced number of communities following the *in silico* permutation. Each hotspot corresponds to the hotspots above it in panels A-D and E-H.

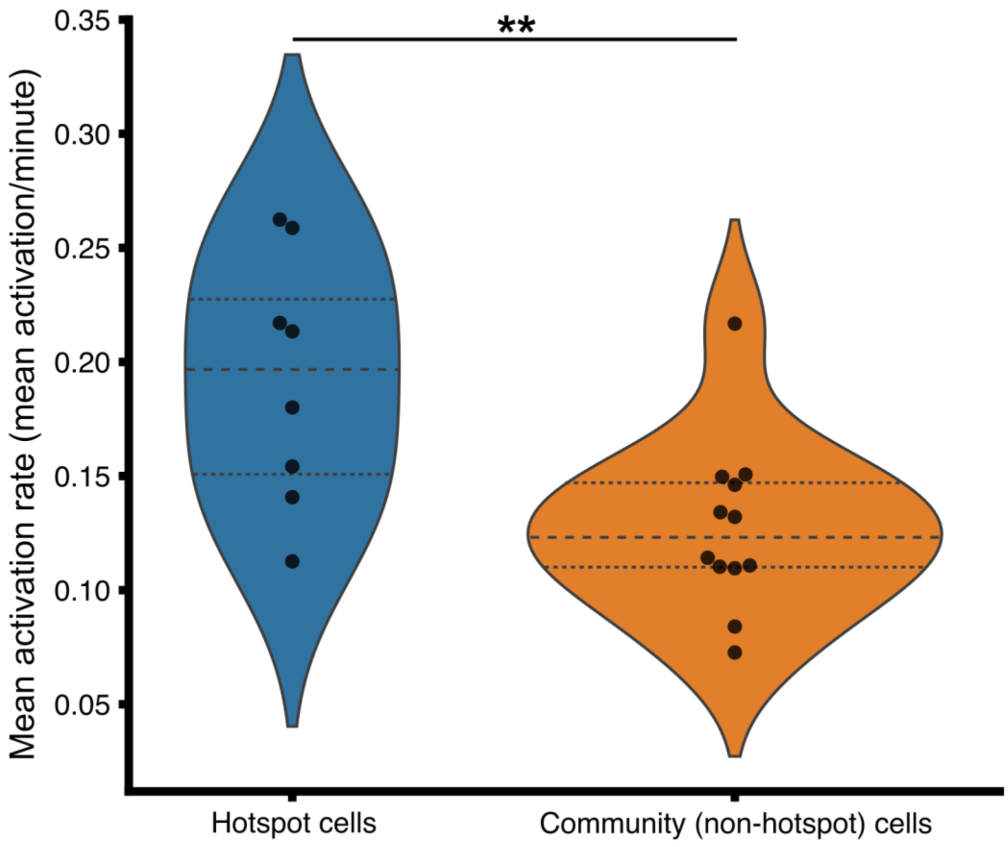

Figure S9. Hotspot cells are more active than community, non-hotspot, cells. Distribution of mean activation rate of hotspots cells (blue) and community (non-hotspot) cells, i.e., cells that participated in transient communities but not in hotspots (orange). For hotspot cells, each black data point denotes the mean activation rate of each statistically significant validated hotspot (N = 8). For community cells, each black data point denotes the mean activation rate of all community and non-hotspot cells in each wild-type LG (N = 12). Kruskal-Wallis statistical test showed significant difference in distributions of activation rate between hotspot cells and community cells (p-value < 0.01). Specifically, we measured an average activations per minute of 0.192 for hotspots cells compared to 0.127 for cells that participated in communities that did not form hotspots. The cells' activation rate was accounted for in the hotspot validation process (see Methods: Hotspots analysis).

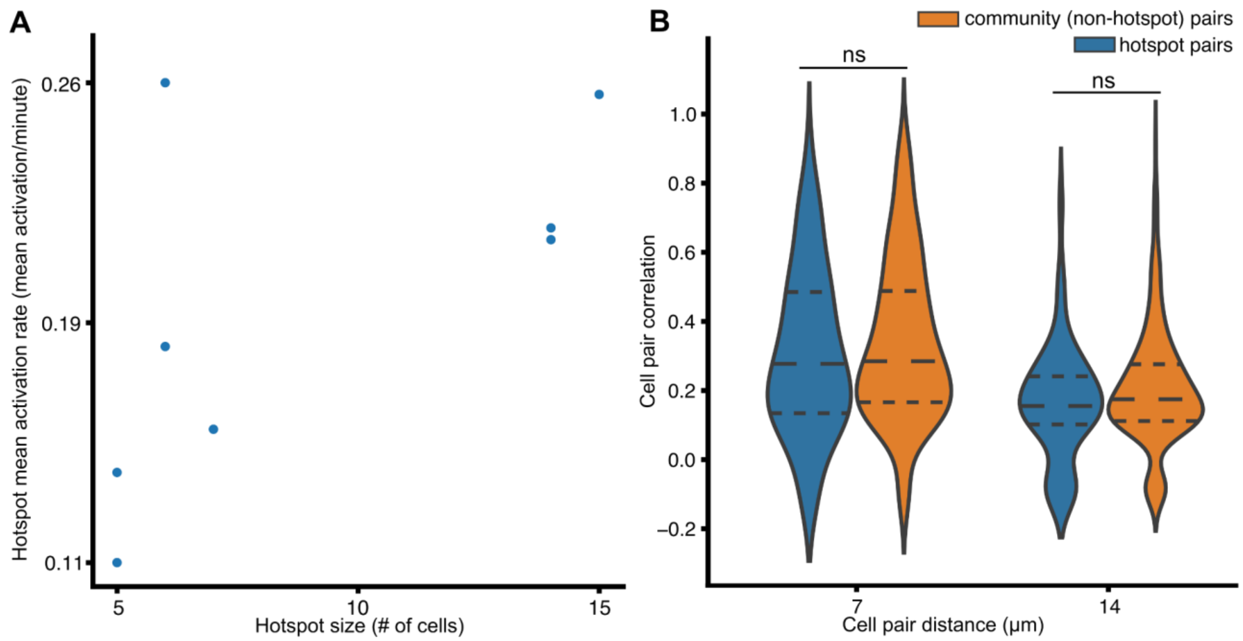

Figure S10. Characterizing hotspots. (A) Each data point reflects the size (x-axis) and the mean activation rate of cells (y-axis) in a single hotspot. N = 8 statistically significant validated hotspots pooled across wild-type LGs. Pearson correlation (coefficient = 0.615, p-value = 0.104) between hotspot size and its activation rate. **(B)** Comparison of temporal Ca^{2+} signal correlation between cell pairs in hotspots to cell pairs that participated in communities (but not hotspots). Maximal correlation between each pair's time series was recorded. Each cell pair was binned according to its distance, with intervals of 7µm, approximately equivalent to 1 cell diameter. We examined pairs of up to 14µm and allowed a time lag of up to 15 seconds, in accordance with the parameter used to define the transient communities (see Methods). Kruskal-Wallis statistical test showed no significant difference in distributions of correlations between hotspot cells and community (non-hotspot) cells in both distance groups (p-value > 0.05).

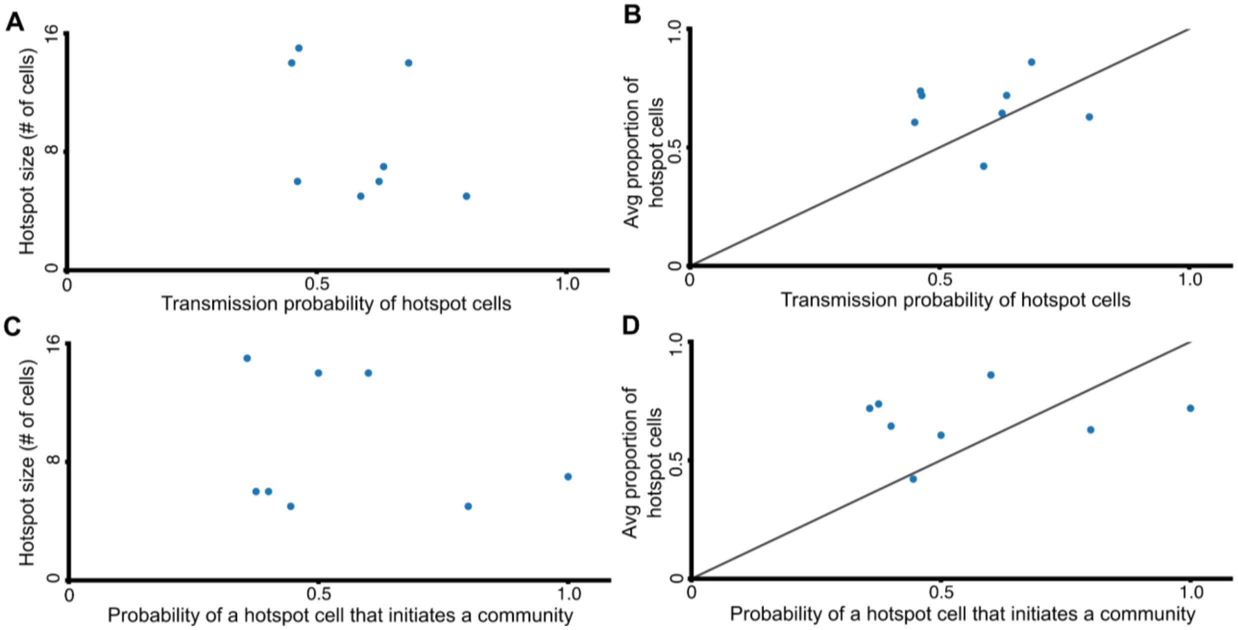

Figure S11. Interactions of hotspots with their surrounding cells did not have a predominant direction. Each data point reflects the probability across all transient communities in a statistically significant validated hotspot. N = 8 hotspots pooled across wild-type LGs. The average proportion of hotspot cells in transient communities (y-axis in B and D) was defined as the mean of the ratio between the number of hotspot cells in a community and the total number of cells in that community. The diagonal (y = x) indicates the situation where the average proportion of hotspot cells corresponds to their probability of transmitting a signal, i.e., activating before non-hotspot cells in the same transient community (B), or initiating a transient community, i.e., activated first in the transient community (D). Transmission probability was calculated from all pairs of adjacent hotspot and non-hotspot cells in a common transient community (see Methods). **(A-B)** Probability of signal transmission from hotspot cells to non-hotspot cells as a function of hotspot size (**A**) and as a function of the average proportion of hotspot cells in transient communities (**B**). **(C-D)** The probability of a hotspot cell to initiate a transient community as a function of hotspot size (**C**) and as a function of the average proportion of hotspot cells in transient communities **(D)**.

Supplemental video titles and legends

Video S1: Ca^{2+} signaling dynamics in blood progenitors of a wild-type LG, visualized using GCaMP6f (green). Timestamp is measured in seconds.

Video S2: Ca^{2+} signaling dynamics of a wild-type LG, highlighting three transient communities (each community is annotated by a red, white, or yellow polygon). Timestamp is measured in seconds.

Video S3: The formation and disintegration of the 6-cell transient community shown in Fig. 1D. Timestamp is measured in seconds.
